# T Cell Membrane Heterogeneity Aids Antigen Recognition and T Cell Activation

**DOI:** 10.3389/fcell.2020.00609

**Published:** 2020-07-28

**Authors:** Megan V. Farrell, Samantha Webster, Katharina Gaus, Jesse Goyette

**Affiliations:** ^1^EMBL Australia Node in Single Molecule Science, School of Medical Sciences, University of New South Wales, Sydney, NSW, Australia; ^2^ARC Centre of Excellence in Advanced Molecular Imaging, University of New South Wales, Sydney, NSW, Australia

**Keywords:** T cells, microvilli, T cell receptor, membrane curvature, membrane heterogeneity, T cell activation

## Abstract

T cells are critical for co-ordinating the immune response. T cells are activated when their surface T cell receptors (TCRs) engage cognate antigens in the form of peptide-major histocompatibility complexes (pMHC) presented on the surface of antigen presenting cells (APCs). Large changes in the contact interface between T cells and APCs occur over the course of tens of minutes from the initial contact to the formation of a large-scale junction between the two cells. The mature junction between a T cell and APC is known as the immunological synapse, and this specialized plasma membrane structure is the major platform for TCR signaling. It has long been known that the complex organization of signaling molecules at the synapse is critical for appropriate activation of T cells, but within the last decade advances in microscopy have opened up investigation into the dynamics of T cell surface topology in the immune synapse. From mechanisms mediating the initial contact between T cells and APCs to roles in the organization of molecules in the mature synapse, these studies have made it increasingly clear that local membrane topology has a large impact on signaling processes. This review focuses on the functional consequences of the T cells' highly dynamic and heterogeneous membrane, in particular, how membrane topology leads to the reorganization of membrane proteins on the T cell surface.

## Introduction

T cells are integral members of the immune system, in which they mediate adaptive immunity through direct, antigen-specific contact with antigen-presenting cells (APCs). In order to initiate an appropriate immune response, the T cell relies upon external signals communicated to them by proteins on the surface of an APC. The signal given to the T cell determines the effector response, for instance, an APC can trigger the activation of a T cell by presenting peptide antigens on major histocompatibility complexes (pMHC). pMHCs can bind to the T cell receptor (TCR) on the T cell membrane, triggering a signaling cascade through the associated cluster of differentiation 3 (CD3) subunits, which includes recruitment of cytosolic signaling proteins such as the protein kinase zeta-chain-associated protein kinase 70 (Zap70) and phosphorylation of membrane proteins such as the linker for activation of T cells (LAT) (Balagopalan et al., 2015; Courtney et al., 2018).

These events lead to the formation of the immunological synapse, a reorganization of molecular components, membrane proteins, and the actin cytoskeleton, which is characteristic of active T cells. In addition to pMHCs, the APC also presents inhibitory ligands, which bind to cognate receptors on the T cell and lead to signal termination and inactivation of the T cell (Chen and Flies, 2013). Spatial reorganization of membrane-bound activating and inhibitory receptors is an essential factor for determining the response of a T cell, with inhibitory receptors being shown to be excluded from the area of activating receptor engagement (Choudhuri et al., 2005; Burroughs et al., 2006). Thus, T cells must be equipped to respond to a diverse range of extracellular signals.

The plasma membrane is the major platform for T cell signaling. For this reason, heterogeneities in the T cell membrane can lead to alterations in protein organization and signaling, and therefore, have functional consequences for the T cell. Heterogeneity in the plasma membrane can be caused by multiple different factors. For instance, changes in membrane topology can cause the plasma membrane to decrease the spatial scale on which protein-protein interactions occur in order to promote weak, yet biologically important receptor-ligand interactions (Choudhuri et al., 2005; Stone et al., 2017). Additionally, intracellular processes, such as the reorganization of the actin cytoskeleton, may influence protein diffusion and compartmentalization within the membrane, as well as regulate the nanoscale clustering of certain membrane proteins (Sadegh et al., 2017). Thus, the study of the organization of receptors and other molecules in the plasma membrane, as well as the dynamics of the plasma membrane itself, can reveal novel insights into the activation of T cells and their function as mediators of the adaptive immune system.

In this review we outline general membrane features and discuss how membrane curvature can influence the distribution of proteins on the membrane. This is followed on by an example of how these processes can affect cellular functions. Finally, we discuss how these highly dynamic and heterogeneous membranes can result in functional consequences for T cells, in particular how membrane topology leads to the reorganization of the membrane proteins on the T cell surface.

## Membrane Features

Membrane topology encompasses the complex and dynamic system of morphological features present at the surface of the T cell membrane. Morphological features, i.e., protrusions and invaginations, of the T cell membrane are naturally suspected to significantly impact the protein distribution and composition at the membrane and therefore are likely to play some role in intracellular signaling and cell function (Chang et al., 2016; Jung et al., 2016; Razvag et al., 2018, 2019; Ghosh et al., 2020). The localization of signaling proteins to either protrusive or intrusive regions of the cell membrane, or conversely, the segregation of signaling molecules from areas of high membrane curvature suggests a functional relationship between cell surface morphology and subcellular signaling.

**Figure 1:**
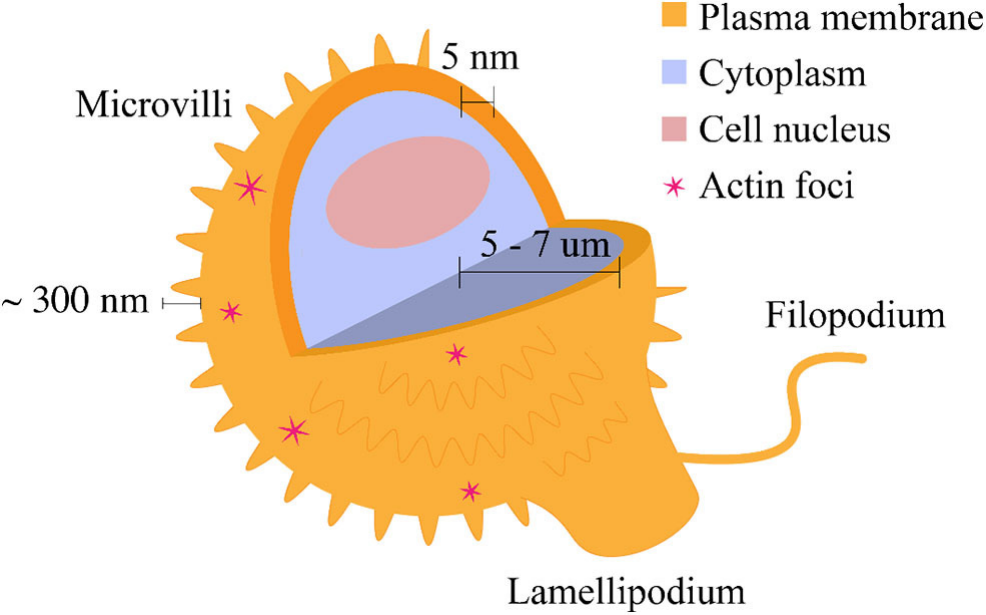
Morphological features of the T cell plasma membrane. The plasma membrane is shaped by the underlying actin cytoskeleton. These morphological features are related to surface protein distribution and consequently, cell signaling. Microvilli cover most of the cell surface and are composed of actin filaments. Lamellipodia and filopodia are related to cell migration and are also composed of actin. Actin foci may be observed in a variety of characteristic shapes (Fritzsche et al., 2017). The features are not depicted to scale.

The T cell membrane is not spatially homogeneous, and in fact there are several morphological motifs present on the cell surface (Figure 1). One major morphological feature of the T cell membrane is the small, cylindrical protrusions which cover the surface of circulating mammalian lymphocytes. These lymphocyte microvilli, or invadosome-like protrusions (ILPs) (Sage et al., 2012), are highly dynamic, actin-based structures which have been shown to continuously assemble and disassemble at the cell surface. The first quantitative characterization of lymphocyte microvilli by Majstoravich et al. used scanning electron microscopy to determine that microvilli contain long, parallel bundles of actin filaments (Majstoravich et al., 2004). These bundles are disrupted by treatment with Latrunculin A, which causes actin end-blocking, suggesting continuous actin assembly and reassembly at the cell surface, changing on a time scale of seconds to minutes (Majstoravich et al., 2004). More recently, Kim et al. used a combination of techniques to determine that T cells generate microvillus-originated particles upon T cell stimulation (Kim et al., 2018).

Small lymphocyte microvilli on T cell surfaces may be used as sensory organs to enable the search for pMHCs (Brodovitch et al., 2013). T cells must efficiently survey the surface of APCs for pMHCs in order for TCR recognition to occur and enact a signaling cascade which mounts an adaptive immune system response (Baumgart et al., 2019). Cai et al. explored the role that active surface topology plays in ligand detection. Their results indicate that TCR recognition appears to happen at the same time as surface deformations provide initial contact. However, it is still unclear whether the initial surface deformation contact zones are stable or whether cells continue to survey the surface of the APC after initial contact (Cai et al., 2017). Characterization of microvilli movement on the T cell reveals an apparent fractal distribution on the surface, which would provide consistent coverage across scales (Cai et al., 2017). Microvilli contacts on a supported lipid bilayer imaged with a lattice light-sheet microscope occurred at consistent densities over time. These dynamic, actin-rich adhesion structures may probe APCs during T cell searching. In one study, the intercellular contacts between the T cell and APC were found to consistently precede and support T cell activation as indicated by increased calcium flux. On antigen-pulsed APCs, T cells rapidly fluxed calcium, lost spatial polarity, and arrested migration and under these activating conditions, a dense array of “podo-prints” largely stably (18 min) localized at the periphery of the T cell/APC interface (Linder, 2009; Sage et al., 2012).

In addition to microvilli, other morphological features include filopodia and lamellipodia, which are involved in cell migration and contribute to the regulation of signaling (Figure 1). While their contribution to migratory cell processes is closely related, these structures differ in their actin content; lamellipodia are sheet-like protrusions which contain branched actin networks, while filopodia are thin structures with tight, parallel bundles of F-actin (Mattila and Lappalainen, 2008). Fritzsche et al. describe actin foci, another morphological feature present on the T cell membrane. Actin foci may be polymerized into self-organized actin patterns (Fritzsche et al., 2017). These self-organized patterns may take the form of actin vortices, asters, or stars (Fritzsche et al., 2017) and can lead to distal signaling events in T cells, including phospholipase C, gamma 1 (PLCγ1) activation and subsequent cytoplasmic calcium ion elevation (Kumari et al., 2015). Table 1 provides a summary of the dimensions of the morphological features discussed above.

**Table 1:** Approximate measurements of T cell membrane features.

**Feature**	**Length**	**Width**	**Proposed function**	**References**
Actin foci			Facilitate distal signaling events in T cells, including PLCγ1 activation and subsequent cytoplasmic calcium ion elevation	Kumari et al., 2015; Fritzsche et al., 2017
Vortices^†^ Asters/stars^†^	– –	500 nm 2.5–5 μm		
Filopodia	5–7 μm	0.1–0.3 μm	Probe environment and assist with cell migration	Mattila and Lappalainen, 2008; Kim et al., 2018
Lamellipodia	–	0.1–0.2 μm	Promote cell migration and extension	Mattila and Lappalainen, 2008
Microvilli or ILPs	0.3–5 μm	0.2–0.5 μm	Scan and search antigens on APC, perhaps also serve as immunological synaptosomes	Majstoravich et al., 2004; Sage et al., 2012; Cai et al., 2017; Kim et al., 2018

*For reference, T cells have a diameter of approximately 6 to 12μm (Rosenbluth et al., 2006)*.

† *Actin vortices, asters, and stars are representative of structures which result from actin self-organization at actin foci. Actin vortices may transition into actin stars and asters*.

## Curvature-Driven Regulation of Protein Distribution

There is no comprehensive explanation for the mechanisms of interaction between cell morphology and signal transduction. However, cell morphological motifs alone can offer a mechanism for the spatial organization of molecular signals at the subcellular scale through co-localization of proteins and morphological structures. Methods are being developed to detect and identify subcellular morphological motifs as well as calculate relevant parameters such as mean curvature, volume, and surface area of structures such as filopodia, lamellipodia, and microvilli (Driscoll et al., 2019). The data may be acquired through both high resolution light-sheet microscopy techniques and more conventional microscopy techniques.

Previously established membrane curvature-sensing models have relied on specific protein structural motifs such as amphipathic or hydrophobic structure insertion, protein scaffolding, Bin/Amphiphysin/Rvs (BAR), and amphipathic helix insertion, or steric interactions to facilitate energetically favorable binding to the membrane (Callan-Jones and Bassereau, 2013). However, disordered proteins also seem to exhibit curvature-sensing mechanisms when binding to the membrane. Several studies suggest that certain proteins, even those without specific curvature-sensing domains, have the capacity to sense and preferentially localize to sites of distinct curvature on the membrane (Bartels et al., 2010; Larsen et al., 2015; Zhao et al., 2017). Zeno et al. present a model that includes both an entropic and electrostatic mechanism to attempt to explain the ability of disordered proteins to find and bind to structural heterogeneities in the plasma membrane (Zeno et al., 2019). The model suggests that as membrane curvature increases, steric hindrance decreases between the disordered protein and the membrane and chain entropy increases, making it entropically favorable for proteins with a high degree of conformational energy to bind to high curvature locales on the membrane. As the curvature increases, the average distance between anionic membrane lipids and negatively charged amino acid chains also increases making this an electrostatically favorable configuration for proteins with high net negative charge. This model could be used to give a conceptual explanation for why certain surface proteins exhibit curvature preference on the plasma membrane. Another model aims to describe the relationship between membrane curvature and transmembrane protein distribution with a purely thermodynamic approach (Aimon et al., 2014). The membrane is modeled as a thin (quasi-2D) fluid film in which proteins freely diffuse and the addition of each protein to this surface alters the bending energy of the membrane. The protein distribution is then determined through a calculation which minimizes free energy.

The models discussed above all aim to describe the mechanisms through which proteins preferentially distribute themselves on the curved plasma membrane. To add context to these models, we now discuss some mechanisms which create curvature gradients on the plasma membrane, and therefore mediate curvature-driven regulation of protein distribution.

Mechanical forces generate curved membrane surfaces which may facilitate T cell activation. One investigation of the mechanical forces generated upon engagement of the TCR and Lymphocyte function-associated antigen 1 (LFA-1) gave a quantified description of a force generation sequence upon local bidimensional engagement of the TCR-CD3 complex (Husson et al., 2011). The sequence is described by an initial latency phase, which is followed by a pushing phase and then a pulling phase. During the pushing phase, the cell extended a directional growth toward the APC. The initial growth of the T cell protrusion toward the APC was characterized by a constant growth speed which is comparable to actin polymerization kinetics, while co-engagement of LFA-1 and TCR showed a clear decrease in the protrusion length emitted in the pushing phase. Another study characterized T cell spreading response as a function of substrate rigidity (Wahl et al., 2019). Depending on the surface receptors stimulated, the cellular response may either be biphasic or monotonous. Actin polymerization-generated forces are the basis for this theoretical description of mechanosensitive spreading of T cells. In the authors' proposed model, the biphasic behavior of the driving tensile force depends on high effective bond stiffness and low bond force sensitivity.

The connection between the capacity for curvature-sensing, which many proteins and peptides exhibit, and the membrane surface protein distribution is not yet well-defined. However, structural mechanisms as well as thermodynamic models can now provide insight into the curvature-driven regulation of protein distribution on the plasma membrane. The processes through which this curvature is created may then also play a major role in the membrane curvature/protein distribution relationship. In effect, cellular mechanisms which induce curvature in the plasma membrane could have several nontrivial consequences for cell signaling processes.

## Membrane Protein Distribution

The mechanism by which cell surface proteins seem to be able to detect changes in membrane curvature is currently a poorly understood phenomenon. However, many studies have shown the redistribution of proteins, such as the TCR, in response to curvature in the cell membrane, particularly favoring localizing on protrusions such as microvilli (Sage et al., 2012; Cai et al., 2017). Protein organization on the T cell membrane has previously been characterized using single molecule localization microscopy (SMLM), showing this spatial reorganization of proteins on the surface of T cells is essential for the TCR triggering, therefore conferring a functional significance for T cells (Pageon et al., 2016).

**Figure 2:**
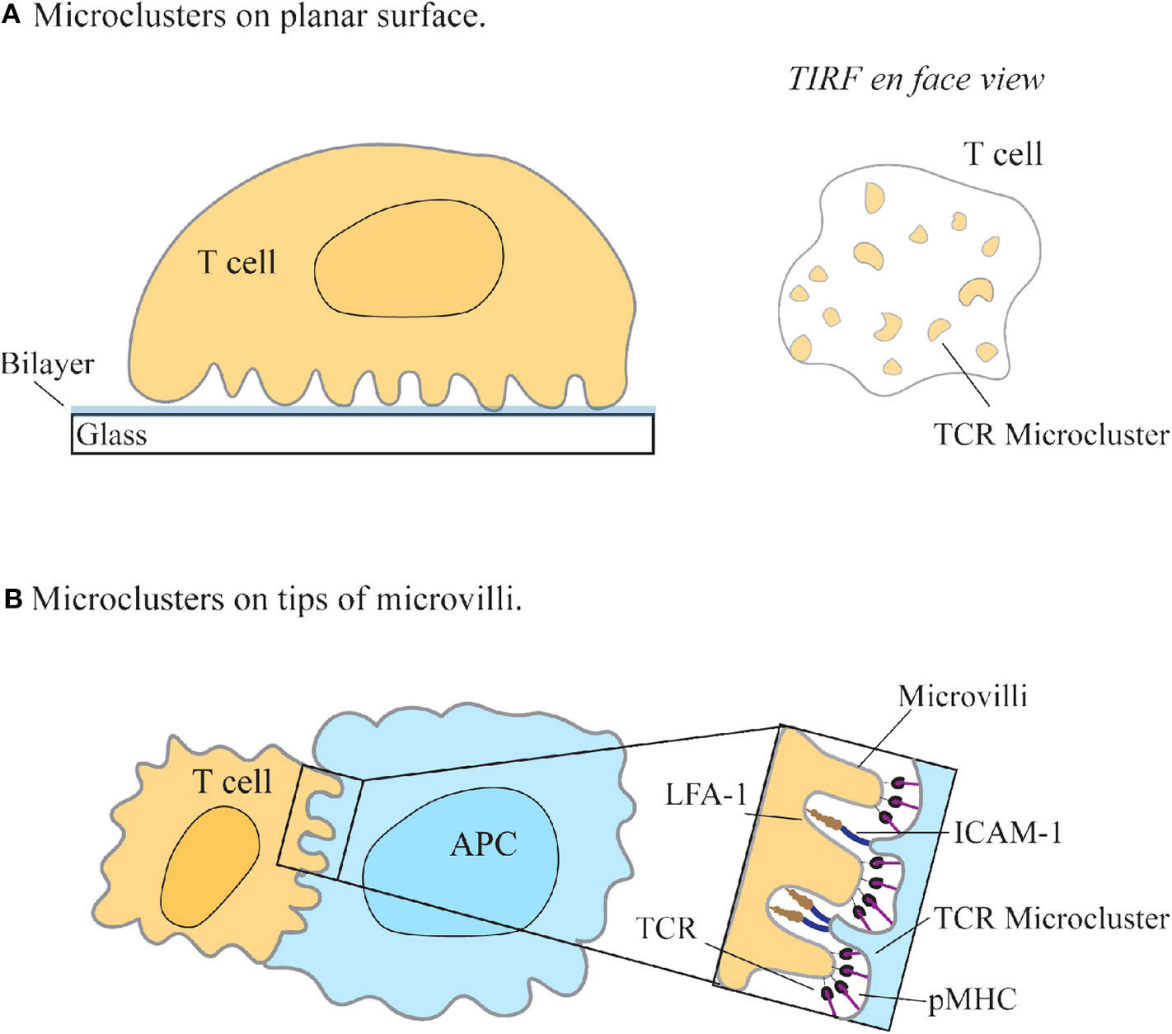
T cell receptor enrichment on microvilli. **(A)** T cell receptors (TCR) seem to redistribute into microclusters upon engagement with a SLB-coated surface using TIRF-microscopy. (**A**, right) *En face* view of the interface between the cell and the coverslip, as seen with TIRF-microscopy. **(B)** Jung et al. found that TCRs preferentially localize to the tips of microvilli on T cells to facilitate searching of antigen presenting cells (APC) for cognate pMHC (Jung et al., 2016). Zoom-in region shows the contact interface with the larger adhesion molecules LFA-1 and ICAM-1 binding at the base and TCR/pMHC interacting at the tips of microvilli (Neve-Oz et al., 2018).

The redistribution of TCRs into small peripheral bundles observed using SMLM were termed micro- or nano-clusters and have since become a defining feature of T cell activation (Figure 2A). This called into question how the reorganization of proteins into spatially heterogeneous clusters on the membrane gives a functional advantage to the cell by facilitating T cell triggering. The answer has been theorized by various activation models. The kinetic proofreading model, together with the induced rebinding model, postulates that the clustering of TCR molecules allows for discrimination between the 10^-4^ lower-levels of antigenic-pMHC to non-stimulating-pMHC molecules, via temporal lags in binding, and rapid rebinding to pMHC molecules (Mckeithan, 1995; Dushek and van der Merwe, 2014; Goyette et al., 2019). Thus, the grouping of TCR complexes into clusters on the membrane may lead to rapid binding and sampling of pMHC by TCR, thereby allowing T cells to maintain sensitivity and discrimination of cognate peptides.

Following the finding that TCR clustering aids activation, further emphasis was placed on the concept that proteins may compartmentalize on the membrane to facilitate biochemical reactions. A combination of imaging approaches revealed that signaling proteins associated with the LAT signalosome form distinct submicrometer-sized physical compartments at the membrane (Su et al., 2016). It was additionally seen that the formation of these LAT compartments may be driven by lipid phase separation (Ditlev et al., 2019). Additionally, bulky phosphatases such as CD45 are sterically excluded from areas of TCR-ligand engagement, thereby shifting the balance toward phosphorylation upon triggering and further strengthening the theory that membrane protein organization facilitates early T cell signaling (Leupin et al., 2000; Chang et al., 2016). This indicates that heterogeneities in the composition of the plasma membrane may be essential for the compartmentalization of proteins and contribute to altered functional outcomes.

Moreover, it has been well-documented using various microscopy techniques, that membrane proteins involved in T cell activation distribute into distinct regions within a “supramolecular activation cluster” (SMAC) and collectively form an immunological synapse with an APC (Monks et al., 1998). By combining this knowledge with TCR microcluster formation, it was hypothesized that these clusters accumulate and stabilize at the middle of the synapse, forming a central hub of activation, with bulky phosphatases pushed to outer areas (Grakoui et al., 1999). Conversely, later studies found that early stage TCR clusters form at the leading edge of an activating T cell and immediately recruit signaling proteins, with later-stage signaling corresponding to the movement of microclusters to the center of the synapse (Bunnell et al., 2002). Furthermore, early TCR signaling molecules such as Zap70 and SLP-76 dissociate from the TCR upon entry into the central area of the SMAC, leading to signal termination (Yokosuka et al., 2005). Collectively, these studies all show that dynamic protein heterogeneity in the membrane can dramatically shift signaling and functional responses.

The organization of proteins into distinct compartments on the membrane was speculated to also facilitate signal termination. Choudhuri et al. showed microvesicles with high levels of TCR molecules budding from the plasma membrane, indicating that the membrane at the central immune synapse has high curvature (Choudhuri et al., 2014). Later studies using live-cell imaging of the T cell-APC interface defined these budded fragments as T cell microvilli-derived particles (TMP), proposing that T cells leave behind TMPs on APC surfaces post-activation (Kim et al., 2018). The relocation of TCR microclusters to the center of the synapse may, therefore, facilitate budding from the membrane, terminating signaling, removing TCR molecules from the T cell surface, and perhaps a means of trans-cellular communication with the APC in the synapse interface in a process termed “trogocytosis” (Kim et al., 2018).

Much of the knowledge of membrane protein distribution and signal regulation of T cells is collected using TIRF microscopy techniques, allowing for the nanoscale detection of membrane proteins. One compromise when imaging nanoscale protein distributions using super-resolution techniques is the necessity for the cell to be immobilized onto planar surfaces, such as antibody-coated glass and supported lipid bilayers (SLB) (Figure 2A). Although SLBs allowed for the mobility of ligand-protein interactions and the movement of receptors into clusters, the use of a stiff surface to activate cells may mechanically “frustrate” cells and restrict essential membrane formations, an aspect that must be considered when interpreting data collected from planar surfaces (Sage et al., 2012). Additionally, studying T cells under resting conditions is an essential baseline for understanding the changes T cells undergo upon activation. Recent studies show that many features seen in “resting” T cells may be artifacts of the coating used to adhere these cells onto a surface, consequently causing non-physiological responses (Ponjavic et al., 2018; Rossboth et al., 2018; Santos et al., 2018).

Thus, the use of various light microscopy techniques have indubitably been an asset to understanding how these complex immune cells function, however, these methods are inherently limiting. Therefore, one should consider how these findings may apply to broader three-dimensional interactions between a T cell and an APC.

## Topology-Driven Protein Distribution

In physiological settings, T cells make contact with deformable surfaces and must penetrate the thick glycocalyx present on cells. Therefore, protein distribution may vary when the T cell comes into contact with an APC (and may differ from that in a reconstituted system). For these reasons, focus has shifted recently to taking membrane topological features into consideration when activating and imaging immune cells.

Various imaging approaches facilitated the finding that membrane protein distribution may be linked to cell topography. One such approach is combining variable-angle TIRF (VA-TIRF) with stochastic localization nanoscopy (SLN) to determine the relationship between TCR membrane distribution and T cell microvilli (Jung et al., 2016). Through this method, it was hypothesized that microvilli may facilitate antigen searching by T cells, with a higher density of T cell receptors found at the tips of microvilli, suggesting that this enrichment of TCR molecules in resting T cells may be a consequence of membrane topological features (Jung et al., 2016) (Figure 2B). This supported the observation, using SMLM techniques, that the TCR complex is pre-clustered in quiescent T cells (Lillemeier et al., 2010). A defining feature of T cells is their ability to search the large surface area of APCs for cognate pMHCs; this finding thus suggested an explanation for how the cell may efficiently facilitate this search.

This discovery shed light on the complicated process of APC searching, however, the method microvilli used to search was still largely unclear. Advances in light microscopy, such as the ability to form a light-sheet, led to greater insight into these 3D processes (Huisken et al., 2004). Lattice light-sheet microscopy is used to investigate how T cells utilize their dynamic surface protrusions to interact with large surface areas of APCs and find cognate pMHC. Studies show that this searching is conducted in a quick, efficient manner, with microvilli stabilizing upon the recognition of pMHC (Cai et al., 2017; Fernandes et al., 2019). This led to a greater understanding of how the high density of TCR molecules on the tips of the microvilli carried out the search and what occurred upon recognition of a cognate antigen.

The ILPs observed by Sage et al. when activating T cells with endothelial cells pulsed with antigen (similar in size and dimension to previously described microvilli) were shown to be used by T cells for probing cells for antigens, much like microvilli, penetrating the surface of planar endothelial cells and facilitating trans-endothelial migration in the absence of antigen. This finding is consistent with the theory that TCRs are accumulated on the tips of T cell protrusions and, additionally, they showed that these peripheral protrusions are not only enriched in TCR components but also with the corresponding signaling molecules, such as Zap70, suggesting active signaling occurring in protrusions (Sage et al., 2012).

In order for T cells to penetrate the thick glycocalyx, some mechanical forces must be generated. As mentioned previously, using biomembrane force probes, T cells were shown to undergo directional “pushing” toward the model APC at a rate of growth comparable to actin polymerization kinetics. This indicates that microvilli may need to “reach out” to engage cognate pMHCs (Husson et al., 2011). Given the comparable length of microvilli and width of the glycocalyx (estimated 200–500 nm for endothelial cells), this may be a functional mechanism for the cell to protrude through the glycocalyx to reach its surface ligands (Table 1) (Reitsma et al., 2007). Additional works highlighted the role of actin in the spatial and temporal dynamics of T cell signaling at the membrane surface. T cells were shown to generate forces against the interacting surface and actin flow was highlighted as the responsible agent for the mechanical force (Colin-York et al., 2019). This underlines a mechanism by which T cells can maintain signaling from TCR microclusters at the tips of microvilli through the force produced by actin polymerization and, additionally sustaining protein compartmentalization via “molecular clutch” interactions between LAT-associated proteins and actin filaments (Ditlev et al., 2019). Collectively, new insights suggest a dynamic relationship between membrane topology and protein distributions on the T cell surface, a process which may be facilitated by the actin network.

A recent study carried out by Ghosh et al. showed that essential components of TCR signaling are pre-organized on microvilli creating a hub of TCR signaling (Ghosh et al., 2020). This study followed on from previous work using combinational VA-TIRFM and SLN techniques, which revealed pre-clustering of TCR molecules at the tips of microvilli in resting T cells (Jung et al., 2016). They additionally show, using the same approach, that TCR components, the co-stimulating molecule CD2 and the co-receptor CD4 also localize to the microvilli region, with over 90% of molecules within protrusion regions. Microvilli were deemed “hubs” of signaling as the kinase Lck and the signaling adaptor LAT were also localized to microvilli regions, indicating the formation of membrane curvature, in the form of microvilli, promotes signaling from the TCR complexes, thereby facilitating functional consequences (Ghosh et al., 2020). Consistent with previous reports, the phosphatase CD45 was seen to be relatively homogeneously distributed on the T cell membrane, with only ~30% of CD45 molecules localized within microvilli regions, further promoting T cell activation (Cai et al., 2017; Fernandes et al., 2019).

3D studies have shed light on how T cells recognize antigens amongst a sea of self-peptides, utilizing membrane protrusions rich in TCR molecules as antigen sensors, rapidly searching APCs, stabilizing and signaling upon antigen recognition. Thus, heterogeneities in membrane topology, composition and protein distribution within the T cell membrane are demonstrated to be essential for the regulation of effector functions in T cells.

## Membrane Heterogeneity Aids T Cell Activation

The cell membrane is a complex entity, the movement and curvature of which is influenced dynamically by a range of factors all affecting and responding to each other. Influential factors include lipid compositions, trans-membrane proteins responses, e.g., curvature-sensing proteins, mechanical forces, contacts with extracellular matrices, and cell-cell interactions. The combination of all of these processes has intracellular effects on the cell that lead to alternate cellular responses. In the context of T cells, there are multiple ways in which heterogeneities in the membrane such as these can effect the T cells initial activation, inter-membrane architecture and ultimately signaling and cell responses. Membrane curvature creates an uneven surface, on which the first interactors of an APC will be proteins located at the tips of protrusions. In T cells, many studies mentioned earlier in the review have described how the T cell utilizes this by enriching protrusions with TCR molecules in order to rapidly search APCs for cognate pMHC (Lillemeier et al., 2010; Jung et al., 2016). Following this, pre-clustering and/or ligand-induced TCR clustering has been described to increase the selectivity and sensitivity of the T cell response by facilitating binding to multiple pMHC molecules on the APC and allow for rapid re-binding events to occur due to high local concentrations of TCR molecules on the membrane. This process can enhance the sensitivity of the T cell to rare antigens upon recognition and sustain the signaling response (Goyette et al., 2019). Additionally to membrane dimensions influencing protein organizations, the converse can also occur. Following TCR triggering, multi-protein complexes form to create a “hub” of signaling. As discussed previously, the LAT signalosome forms in response to TCR triggering (Su et al., 2016). This signalosome is made up of multivalent binding interactions which coagulate into clusters of intracellular signaling proteins and form “islands” of signaling in the membrane, influencing membrane compositions and rapidly signaling intracellularly, resulting in cellular responses (Ditlev et al., 2019).

Interactions between cells can have profound influences on the heterogeneity of the membrane. Steric and other external biophysical factors can alter protein distributions and induce curvature in the membrane, ultimately having an influence on cellular functions. For instance, the relative size and shape of receptors on the T cell surface, and their associated ligands, are well characterized and have functional consequences at contact interfaces. This knowledge allows for the prediction of how close together the T cell membrane must be to the APC membrane in order for contact to occur. For this reason, heterogeneities in the topology of the membrane help to facilitate the ligation of proteins on the T cell surface, which have functional consequences. Dimensional heterogeneities such as these can have functional impacts on T cell recognition. Adhesion molecules, such as CD2, on T cells, bind to their ligands to create a close contact area between the two cell membranes at a distance close enough (~10 nm) to allow the TCR to bind to the pMHC molecule on the APC, and have been seen to localize to the tips of microvilli (Jung et al., 2016). Additionally, other adhesion molecules, such as LFA-1 and ICAM-1, on T cells and APCs, respectively, create a larger inter-membrane distance (~37 nm) where surface proteins that make up the lymphocytic glycocalyx (rich in sialic acid), such as CD43 and CD45, are excluded (Springer, 1990; Davis and van der Merwe, 2006; Razvag et al., 2019). This proceeds in limiting the lateral mobility of membrane proteins with large extracellular domains through the creation of a physical barrier. Collectively, this creation of ‘tight contact areas' promotes the recognition of antigens by T cells, segregates phosphatases such as CD45 from tight contacts, enriching and laterally trapping them in areas of high local curvature in the membrane, enhancing signaling and sustaining T cell activation (Razvag et al., 2019). Thus, there is dynamic interplay between membrane topology and protein distributions which contribute to the diverse functional outcomes of T cells.

## Conclusion

The interplay between spatial organization of membrane proteins and membrane topological features, i.e., the characteristic heterogeneities of the T cell membrane, has functional consequences and significantly contributes to the fate of T cells. In this review we have covered research which demonstrates that the membrane has heterogeneous processes which affect T cell function. The dimensions of the morphological features which give the membrane biophysical properties, consequently allowing pre-clustering of proteins on the membrane surface, directly affect the organization of membrane proteins at the T cell/ APC contact interface, and though the study of these “initial” protein distributions is currently an active field of research, there remains the critical question of how these clusters are functionally advantageous and to what extent they increase the selectivity and sensitivity of the T cell. Reorganization of membrane proteins at the immunological synapse comes as a result of ligand-induced clustering, which in turn shapes the membrane itself. Thus, the reciprocal relationship between these classifications of T cell heterogeneity—protein pre-clustering and ligand-induced protein clustering—is that protein pre-clustering, due to biophysical mechanisms which determine the membrane shape, defines the protein distribution at the T cell/APC contact interface, which in turn shapes the membrane itself. We predict that this will continue to be an active area of research as more evidence is discovered to demonstrate the intrinsic relationship between membrane heterogeneity and T cell antigen recognition and subsequent activation.

Although there have been huge advancements in investigating the effect of these membrane heterogeneities on T cell activation in recent years, there are still essential questions, to which, answers are yet to be discovered. Reports of the length of microvilli seem to range from 0.3 to 5 μm (Table 1) but is there a functional reason or cause for this variation? Is it simply a matter of standardizing morphological classification? The answer to this may lie in the further characterization and identification of these subcellular morphological motifs, defining criteria such as surface area, volume, and degree of curvature of protrusions seen on the T cell surface. The capacity for curvature-sensing by proteins involved in the initial engagement between the T cell and the APC and the degree to which it affects membrane protein distribution and curvature is also not well-defined. Thus, further investigations are needed to conclude if these surface proteins could amplify or regulate membrane bending. Additionally, it is possible that the formation of these protrusions alters depending on the interacting substrate—these alterations including synthetic activation using coverslips or simply differences in the APC cell type (Sage et al., 2012; Wahl et al., 2019). Furthermore, it has been reported that microvilli aid searching of the APC surface, however, it is still unclear if this searching continues prior to initial contact with a cognate antigen. It also remains unclear whether TCR molecules are pre-clustered at the tips of microvilli, or if clustering occurs upon TCR triggering, as previously described. The answer to these questions lies in further studies, utilizing the recent advances in microscopy, such as high resolution light-sheet microscopy, and continued creative use of conventional techniques to help unveil the hidden intricacies of the T cell/APC interaction interface.

## Author Contributions

MF and SW wrote the manuscript and created the figures. KG and JG provided guidance and edited the manuscript. All authors contributed to the article and approved the submitted version.

## Conflict of Interest

The authors declare that the research was conducted in the absence of any commercial or financial relationships that could be construed as a potential conflict of interest.
